# Sex-Related Differences in the Pharmacological Response in SARS-CoV-2 Infection, Dyslipidemia, and Diabetes Mellitus: A Narrative Review

**DOI:** 10.3390/ph16060853

**Published:** 2023-06-07

**Authors:** Adelina Lombrea, Mirabela Romanescu, Narcisa Jianu, Minodora Andor, Maria Suciu, Dana Emilia Man, Corina Danciu, Cristina Adriana Dehelean, Valentina Buda

**Affiliations:** 1 Doctoral School, “Victor Babeş” University of Medicine and Pharmacy, 2 Eftimie Murgu Street, 300041 Timisoara, Romania; adelina.lombrea@umft.ro (A.L.); mirabela.romanescu@umft.ro (M.R.); narcisa.dinu@umft.ro (N.J.); 2 Research Center for Pharmaco-Toxicological Evaluation, “Victor Babeş” University of Medicine and Pharmacy, 2 Eftimie Murgu Street, 300041 Timisoara, Romania; suciu.maria@umft.ro (M.S.); cadehelean@umft.ro (C.A.D.); buda.valentina@umft.ro (V.B.); 3 Faculty of Medicine, “Victor Babeş” University of Medicine and Pharmacy, 2 Eftimie Murgu Street, 300041 Timisoara, Romania; man.dana@umft.ro; 4 Multidisciplinary Heart Research Center, “Victor Babeş” University of Medicine and Pharmacy, 2 Eftimie Murgu Street, 340001 Timisoara, Romania; 5 Faculty of Pharmacy, “Victor Babeş” University of Medicine and Pharmacy, 2 Eftimie Murgu Street, 300041 Timisoara, Romania; 6 Ineu City Hospital, 2 Republicii Street, 315300 Ineu, Romania

**Keywords:** differences in pharmacological response, pharmacodynamics, adverse drug reactions, lipid-lowering agents, COVID-19, antidiabetic drugs

## Abstract

Pharmacological responses vary by sex in several illnesses. This narrative review summarizes sex variations in pharmaceutical response in SARS-CoV-2 infection, dyslipidemia, and diabetes mellitus. Infection with SARS-CoV-2 is more severe and deadly in men than women. This may be attributed to immunological responses, genetics, and hormones. Some research shows that men may respond better to genomic vaccinations and females to antiviral medications such as remdesivir (Moderna and Pfizer-BioNTech). In dyslipidemia, women tend to have greater HDL-C and lower LDL-C than men. Some studies show that females may need lower statin dosages than men to obtain equal LDL-C reductions. Ezetimibe co-administered with a statin significantly improved lipid profile indicators in men compared to women. Statins reduce dementia risk. Atorvastatin decreased dementia risk in males (adjusted HR 0.92, 95% CI 0.88–0.97), whereas lovastatin lowered dementia risk in women (HR 0.74, 95% CI 0.58–0.95). In diabetes mellitus, evidence suggests that females may have a higher risk of developing certain complications such as diabetic retinopathy and neuropathy, despite having lower rates of cardiovascular disease than males. This could be the result of differences in hormonal influences and genetic factors. Some research shows females may respond better to oral hypoglycemic medications such as metformin. In conclusion, sex-related differences in pharmacological response have been observed in SARS-CoV-2 infection, dyslipidemia, and diabetes mellitus. Further research is needed to better understand these differences and to develop personalized treatment strategies for males and females with these conditions.

## 1. Introduction

The investigation of sex disparities has a relatively recent history in clinical medical practice, clinical trials, and academic research [1]. It has been shown that the epidemiology, etiology, clinical signs, and consequences of a variety of medical disorders vary by gender and sex [2,3,4,5]. Accordingly, the significance of analyses based on sexual orientation and gender has become progressively noticeable in the scientific literature. In 2016, for instance, the US National Institutes of Health introduced awards to encourage the incorporation of sex as a biological parameter in clinical trials. In addition, the European Commission included sex- and gender-specific considerations in its research and innovation initiative, which went into force in 2015–2016. The regulations and guidelines not only encouraged women’s involvement in research and sex-specific analyses, but also addressed gender equality issues [6,7]. 

The term “sex” pertains to the biological distinctions that exist between males and females, encompassing chromosomal, genetic, hormonal, and anatomical disparities. The classification of sex typically involves the categorization of individuals into male, female, or intersex categories. The term “gender” pertains to the identities of individuals that are shaped by socially established roles, cultural norms, behaviors, and expressions [8]. Sex and gender are both significant factors in health and disease, operating distinctly and influencing an individual’s health outcomes throughout their lifespan. Although sex and gender are distinct concepts, it can be difficult to disentangle them [6]. It is increasingly acknowledged that research review by research ethics committees (RECs) demands the application of a gender perspective. This entails the need for gender training of RECs and the attainment of gender parity in their membership [9]. Despite the lack of empirical evidence, it is probable that an REC that exhibits gender balance would approach issues from a distinct perspective and demonstrate a heightened awareness of gender-related concerns. The under-representation of women on ethics committees is a prevalent issue, which can be attributed to a dearth of national and local laws and regulations, or structural factors such as discriminatory demographic trends [10]. These trends have resulted in a lower representation of women in higher echelons of science and medicine. Up-to-date information is required to determine whether there has been an increase in gender parity within ethics committees and whether there is now a greater emphasis on acquiring knowledge and proficiency in gender-related matters [11].

Extensive research in rodents, humans, and other animals has emphasized the impact of sex dissimilarities on metabolic disorders, such as diabetes, obesity, and cardiovascular diseases (CVDs) [12,13,14,15]. Sex-related differences may be caused by variability in hormonal effects and in the expression of genes encoded in X and Y chromosomes [16,17]. The sexual hormones and the sex chromosome complement are accountable for sex-specific differences in body fat distribution, regulation of glucose, insulin regulation, aberrant fat accumulation, and metabolism of lipids. Furthermore, tissue-specific gene regulation varies between men and women, which contributes to metabolic differences [17,18]. 

There are noteworthy dissimilarities between males and females with regard to the bioavailability, distribution, metabolism, and elimination of medication. Sex-based differences can have varying impacts on the effectiveness and safety of drugs, as certain medications may exhibit superior efficacy in either males or females [5,19]. This is of significant importance, especially in the context of prolonged treatment duration. The sex-related disparities in pharmacokinetics and pharmacodynamics can be attributed to physiological differences, such as hormonal regulation and body fat makeup [20]. Furthermore, notable variations exist in terms of the physiology of organs such as the stomach, liver, lungs, and kidneys. On average, there exists a 0.5 unit increase in the pH of gastric juice in women compared to men [21,22]. Additionally, the rate of gastric passage is inversely correlated with the concentration of estrogen. The hepatic mass and perfusion of the organ exhibit a lower magnitude in females as compared to males [23,24]. 

The present article is part of a series of narrative reviews we are currently working on (one being already published) in order to have up-to-date information regarding the latest sex-induced discrepancies in the pharmacological response of high-impact diseases (leading to severe, long-term complications) and the most prescribed classes of drugs [4]. The objective of this narrative review is to evaluate the influence of sex on the effectiveness and safety of prescribed medications utilized for the management of SARS-CoV-2 infection, dyslipidemia, and diabetes mellitus. The purpose is to furnish significant insights that can facilitate the administration of personalized treatment to individuals who are most likely to derive optimal benefits.

## 2. Results

### 2.1. COVID-19

Females and males differ in their vulnerability and reaction to viral infections, as sex disparities were observed in the prevalence and severity of infectious diseases [25]. Regarding the adaptive immune response, females typically display a greater humoral and cell-mediated immune response to antigenic stimulation, vaccine, and infection [26]. They also show a greater activation of cytotoxic T cells and an upregulated expression of antiviral and proinflammatory genes, both of which have elements of estrogen response in their promoter regions [27]. Conversely, findings regarding the novel coronavirus disease (COVID-19) suggest that men are more affected by SARS-CoV-2 infection, with higher percentages of hospitalization, morbidity, and mortality [28,29]. It has been shown that androgens regulate the expression of TMPRSS2, which is the most commonly altered gene involved in primary prostate cancer and a crucial factor in allowing cell infection by coronaviruses, such as SARS-CoV-2 [30]. Many studies have hypothesized the impact of anti-androgens in male patients with COVID-19, but only a few confirmed this theory in clinical studies [31,32]. According to a prospective cohort study conducted by Goren et al., 15.6% of male subjects who were taking anti-androgens (dutasteride, finasteride, or spironolactone) for at least 6 months until being hospitalized presented a lower risk of admission to the intensive care unit (ICU) than those who were not. There was a slightly smaller proportion of participants admitted to the ICU taking anti-androgens, 8%, versus 58% of those not taking anti-androgens [33] (Table 1). A retrospective cohort study by McCoy et al. analyzed the clinical symptoms of 48 male patients diagnosed with COVID-19 and androgenetic alopecia, for which some of them were using dutasteride as a 5-*α*-reductase inhibitor. The results have shown an important decrease in the incidence of 20 of the 29 clinical symptoms in male patients using dutasteride, with the highest impact on the prevalence of anosmia, ageusia, headache, and dry cough [34]. Clinical studies on the therapeutic action of exogenous estrogen treatment in both female and male patients are presumptive [35,36]. Interestingly, a Phase II clinical study, conducted by Dr. Sharon Nachman from Stony Brook University, is attempting to determine how a transdermal patch that contains 100 µg estradiol can reduce the severity of COVID-19 symptoms compared with standard care in COVID-19-positive patients. They speculated that if administered before intubation, estradiol would decrease the severity of symptoms in older men and women [37]. In a scoping review, Schiffer et al. concluded that there was no sex-stratified randomization in any of the thirty clinical trials on the pharmacological treatment of COVID-19. Just one study stratified its findings through post hoc analysis based on sex [38]. Beigel et al. noted a recovery rate ratio (RRR) for remdesivir, displaying 1.31 (95% confidence interval (CI), 1.07–1.59) RRR for men and 1.38 (95% CI, 1.05–1.81) RRR for women [39] (Table 1). Caruso et al. have recently published a comprehensive review regarding the impact of age and sex on the fatality rate associated with COVID-19. The literature review reveals that women exhibit greater resilience, as evidenced by their longer lifespan during times of severe famines and epidemics, although there is inconsistent evidence regarding centenarian men. Regardless of this, centenarians as a whole do not exhibit a decreased mortality rate compared to other older individuals, likely due to their frailty. Notably, during the initial wave of the 2020 pandemic, centenarians born prior to 1919 displayed greater resilience to COVID-19 compared to younger centenarians, possibly due to their exposure to the 1918 Spanish flu epidemic, although the underlying mechanisms remain ambiguous [40]. Among the numerous therapeutic and preventive treatments established to manage the COVID-19 pandemic, passive immunotherapy with immunoglobulin G (IgG) isolated from the plasma of healthy individuals has been shown to be successful when delivered at the onset of the symptoms. Intravenous IgG exerts its therapeutic effects in autoimmune conditions through a variety of non-specific pathways that target proinflammatory immune reactions [41]. Yet, there is a paucity of evidence on sex-stratified data in immunoglobulin treatment. The comparison study performed by Zeng et al. showed that female patients produce a higher amount of SARS-CoV-2 IgG antibody than male patients in the severe stage of COVID-19 infection. However, although SARS-CoV-2 IgG antibody levels are often greater in female patients than in male patients in the first two to four weeks following illness manifestation, the difference in antibody yield dissipates after four weeks. The authors suggest that monitoring IgG levels may be a useful technique for predicting COVID-19 infection [42]. On the other hand, several studies have documented adverse events after immunoglobulin therapy. For instance, male patients are more likely to develop dermatological diseases, and they may have an unfavorable response to transfusions from female donors, particularly those with a history of pregnancy [43]. Atopy refers to a hyperactive immune response mediated by IgE antibodies against foreign antigens. This response is characterized by metabolic irregularities in the leukotriene pathway, which are of significant importance. Recent research has indicated that sex is a significant factor in the biosynthesis of leukotrienes (LTs), which may partially account for the improved symptom control observed in women with atopic conditions who receive anti-LT medication. Furthermore, the occurrence of diversity in LT production is frequently linked to single nucleotide variations within the arachidonate 5-lipoxygenase (ALOX5) gene. This gene encodes the enzyme system responsible for synthesizing leukotrienes, namely 5-lipoxygenase (5-LO). Mirra et al. conducted a study with the objective of examining the potential involvement of two single nucleotide polymorphisms (SNPs) of ALOX5 in the manifestation of sex differences in allergic disorders. The study was carried out on a prospective cohort of 150 atopic and healthy subjects who were matched based on age and sex. The genotyping of Rs2029253 and rs2115819 was conducted through the utilization of allele-specific RT-PCR. Additionally, the serum levels of 5-LO and LTB4 were quantified using ELISA. The prevalence of both polymorphisms is notably higher in females compared to males. Furthermore, their impact on LT production exhibits a sex-dependent pattern, resulting in a decline in 5-LO and LTB4 serum levels in males and an elevation in females [44]. The pathogenesis of COVID-19 involves immune dysfunction and cytokine storm, as evidenced by various studies [45,46]. Some researchers have reported that vitamin D3 can ameliorate the symptoms of SARS-CoV-2 infection by modulating lung function and acting on the immune system [46]. Additionally, some researchers have proposed that vitamin D3 supplementation may potentially lower the risk of SARS-CoV-2 infection. Gallelli et al. conducted a multicenter cross-sectional study to assess the variations in 25(OH)D3 serum levels among adult individuals who were tested for SARS-CoV-2. The study population comprised acute COVID-19 patients, individuals who had recovered from COVID-19, and non-infected subjects, with a total sample size of 117. The study revealed a statistically significant variance in serum 25(OH)D3 levels among acute COVID-19 patients, with the lowest levels observed (9.63 ± 8.70 ng/mL) in comparison to no-COVID-19 patients (15.96 ± 5.99 ng/mL, *p* = 0.0091) and healed COVID-19 patients (11.52 ± 4.90 ng/mL, *p* > 0.05). The study’s results suggest that the administration of 1α,25(OH)2D3 may be a beneficial treatment for male patients with acute COVID-19 infection. Upon admission, male patients with acute COVID-19 exhibited high levels of circulating IL-6 (139.28 ± 48.95 ng/mL), which significantly decreased following the administration of 1α,25(OH)2D3 (2.65 ± 0.92 ng/mL). Similarly, female patients with acute COVID-19 also exhibited high levels of circulating IL-6 (127.64 ± 22.24 pg/mL) upon admission, which significantly decreased following the administration of 1α,25(OH)2D3 (1.84 ± 0.77 pg/mL) [47].

The pandemic of COVID-19 simultaneously increased the worldwide demand for prophylactic measures (i.e., vaccination), which swiftly became a priority for governments, academia, and the pharmaceutical sector. Vaccines were approved for emergency use, and vaccination efforts began less than a year after COVID-19 had been designated a pandemic [48]. As indicated by clinical studies, males and females show significant variability in their immunological responses to illnesses and vaccines: women often develop a stronger immune response, with antibody titers up to double than those of men [26,49]. The molecular processes behind the sex bias in vaccination immunological response are mostly dependent on sex hormones and genetic/epigenetic factors that influence immune cells [50]. However, data on the disparities in women’s and men’s reactivity to COVID-19 vaccines are limited. The results of the preliminary trials have acknowledged that men had a greater effectiveness rate for the two genomic vaccines: Pfizer 96.4% vs. 93.7% and Moderna 95.4% vs. 93.1% [51,52] (Table 1). Concerning the adenoviral vector-based vaccine Sputnik, the trial’s clinical findings indicated that men had stronger immune responses than women (94.2% in males, 87.5% in females) [53] (Table 1). The AstraZeneca adenoviral-based vaccination elicited equivalent humoral and cell-mediated immune responses in both men and women. Studies examining the adverse effects of COVID-19 vaccines have reported a significantly increased trend among females [54,55] (Table 1). A serious adverse event seems to be linked to AstraZeneca and Janssen vaccines: very uncommon thrombosis and thrombocytopenia, mostly in women under the age of 60 [56]. In line with the aforementioned studies, Heidari and co-workers have published a compelling systematic review based on the existing results of interventional and observational clinical trials. The review provided fresh insight into sex differences regarding COVID-19 vaccines. The assessment of the sex-disaggregated trials has established that the efficacy of COVID-19 vaccines does not show significant disparities between males and females [57]. Concerning the adverse events following immunization with COVID-19 vaccines, the published data indicate statistically significant variations by sex. Females are prone to vaccine reactions, especially anaphylaxis after the administration of Moderna or Pfizer-BioNTech vaccines [58,59] (Table 1). In contrast, a 2022 survey of 8269 healthcare professionals revealed that women who were vaccinated with Moderna or AstraZeneca experienced greater reactogenicity (headache, chills, fever, malaise, rash, gastrointestinal disorders, etc.) than men (odds ratio (OR) 0.66, 95% CI 0.58–0.75, *p* < 0.001) [2] (Table 1). A vigorous debate was raging over the relationship between pharmacological treatment for CVD and COVID-19 severity, and the likelihood that these medications may increase the severity of the infection. In this light, Ma et al. sought to identify sex variations in the liaison between renin–angiotensin–aldosterone system inhibitors and unfavorable outcomes in COVID-19 patients. Among 77,221 UK Biobank participants (53.5% women), the use of angiotensin-converting enzyme inhibitors induced a potential mortality risk for male COVID-19 patients (OR 1.15, 95% CI 1.01–1.32), but calcium channel blockers exhibited a protective activity (OR 0.87, 95% CI 0.79–0.96). The proposed mechanism may be that decreased calcium entrance into cells might alter crucial phases in the viral life cycle. In contrast, the use of angiotensin-receptor blockers was associated with a reduced incidence of COVID-19 death in female patients (OR 0.67, 95% CI 0.47–0.96) [60] (Table 1).

**Table 1:** Summary table of the publications that are part of this narrative review pertaining to COVID-19.

Study Reference	Study Design	Summary of Findings
Goren, A. et al. Anti-Androgens May Protect against Severe COVID-19 Outcomes: Results from a Prospective Cohort Study of 77 Hospitalized Men (2020) [33]	prospective cohort study	Anti-androgens (dutasteride, finasteride, or spironolactone) used for 6 months before hospitalization reduce the chance of intensive care unit admission
Beigel, J. et al. Remdesivir for the Treatment of Covid-19—Final Report (2020) [39]	double-blind, randomized, placebo-controlled trial	Female patients treated with remdesivir exhibited a higher recovery rate ratio than male patients
Baden, L. et al. Efficacy and Safety of the MRNA-1273 SARS-CoV-2 Vaccine (2020) [51] Polack, F. et al. Safety and Efficacy of the BNT162b2 MRNA Covid-19 Vaccine (2020) [52] Logunov, D. et al. Safety and Efficacy of an RAd26 and RAd5 Vector-Based Heterologous Prime-Boost COVID-19 Vaccine: An Interim Analysis of a Randomised Controlled Phase 3 Trial in Russia (2021) [53]	double-blind, randomized, placebo-controlled trials	Genomic and adenoviral vector-based vaccinations such as Pfizer, Moderna, and Sputnik were more effective in men than women
Ewer, K. et al. Cell and Antibody Responses Induced by a Single Dose of ChAdOx1 NCoV-19 (AZD1222) Vaccine in a Phase 1/2 Clinical Trial (2021) [54]	randomized controlled trial	Both men and women responded similarly to the AstraZeneca adenoviral-based immunization
McMahon, D.E. et al. Cutaneous Reactions Reported after Moderna and Pfizer COVID-19 Vaccination: A Registry-Based Study of 414 Cases (2021) [58] Shimabukuro, T. et al. Allergic Reactions Including Anaphylaxis after Receipt of the First Dose of Pfizer-BioNTech COVID-19 Vaccine (2021) [59]	registry analysis	Females are more likely to experience anaphylaxis following Moderna or Pfizer-BioNTech vaccines
Nachtigall, I. et al. Effect of Gender, Age and Vaccine on Reactogenicity and Incapacity to Work after COVID-19 Vaccination: A Survey among Health Care Workers (2022) [2]	survey	Women who received Moderna or AstraZeneca vaccines had more reactogenicity (headache, chills, fever, malaise, rash, gastrointestinal issues, etc.) than males
Ma, Y. et al. Sex Differences in Association between Anti-Hypertensive Medications and Risk of COVID-19 in Middle-Aged and Older Adults (2021) [60]	registry analysis	Angiotensin-receptor blockers decreased COVID-19 mortality in female patients

### 2.2. Dyslipidemia

Dyslipidemia is a crucial component in the pathogenesis of acute coronary syndrome, given that low-density lipoprotein cholesterol (LDL-C) retention in the artery wall initiates atherogenesis [61]. Decreased LDL-C levels have been linked with a reduced risk of subsequent cardiovascular events [62]. Statins are recommended as the first-line therapy for hypercholesterolemia and the prevention of CVD due to their capacity to decrease LDL-C levels via 3-hydroxy-3-methyl-glutaryl-CoA (HMG-CoA) reductase inhibition. They also possess pleiotropic effects manifested by slowing down the progression of atherosclerosis and suppressing the mechanisms of inflammation. Moreover, they facilitate a decrease in oxidative stress and an improvement in antioxidant defense [63] and also demonstrate efficacy in reversing endothelial dysfunction, regardless of the decrease in cholesterol levels [64]. Studies have shown that men often have a more atherogenic plasmatic lipid profile than premenopausal women [65,66]. Premenopausal women have accelerated LDL-C clearance relative to men, resulting in lower LDL-C plasmatic concentrations [67]. In addition, women have increased rates of apolipoprotein A-I synthesis resulting in higher concentrations of high-density lipoprotein cholesterol (HDL-C) [68]. These sex-specific variations in plasma lipid profile are known to be substantially mediated by endogenous sex hormones, which are essential modulators of lipid metabolism [66]. Available data indicate that women diagnosed with coronary artery disease are consistently less likely to receive cholesterol-lowering medication compared to men (59.0% of women and 71.5% of men) [69]. Interestingly, in the clinical trials involving statins, women are underrepresented. Controversial debates question the merits of statins in women due to the assumption that women are secured from cardiovascular disorders until menopause [70] (Table 2). Recent studies focus on sex-linked disparities in treatment and response to statins regarding plasmatic lipid levels and primary and secondary cardiovascular prevention [71,72]. Karlson et al. determined the correlation between age and gender and the administration of rosuvastatin 5–10 mg, atorvastatin 10–80 mg, and simvastatin 10–80 mg among 3155 patients with dyslipidemia and cardiovascular risk. The results of the meta-analysis have shown that all patient groups had substantial dose-dependent decreases in LDL-C and increases in HDL-C. Compared with men, women presented a 2.1% greater decrease in LDL-C (*p* < 0.0001). The authors attributed the result to endogenous sex hormones and their activity as regulators of lipoprotein metabolism. Moreover, the impact of statin therapy on LDL-C and HDL-C was superior in patients over 70 years of age. This fact may be attributed to pharmacokinetic variations between the two groups, but further investigations are warranted [73] (Table 2). Sex-specific differences among female patients treated with maximally intensive doses of rosuvastatin (40 mg) were underlined by a randomized controlled trial (RCT) carried out by Puri et al. They reported a greater percent atheroma volume regression and total atheroma volume regression among women treated with rosuvastatin compared to their male counterparts: −10.1 ± 1.1 mm^3^ vs. −7.16 ± 0.65 mm^3^, *p* = 0.023). In addition, women also presented higher levels of HDL-C: 54.3 ± 12 mg/dL vs. 47.6 ± 11 mg/dL, *p* < 0.001 [70] (Table 2). The management of residual and persistent CVD risk in statin-treated patients has been established as a critical preventative approach. CVD risk reduction has been proven using low-density lipoprotein cholesterol-targeting therapies, with equivalent results among both males and females. According to a meta-analysis of 27 RCTs (174,000 participants, 47,000 women), the proportionate reductions per 10 mmol/L decrease in LDL cholesterol in major vascular events were comparable for women and men (rate ratio (RR) 0.84, 99% CI 0.78–0.91, adjusted *p*-value for heterogeneity by sex = 0.33). Similarly, there were no significant sex differences in the proportionate reductions in major coronary events, coronary revascularization, and stroke. As a result of these cumulative benefits, statin treatment resulted in lower all-cause death rates for both men and women (adjusted heterogeneity *p* = 0.43; RR 0.91, 99% CI 0.84–0.99) [74]. Based on the fact that highly polymorphic hepatic cytochrome P-450 is responsible for the metabolism of all main statins, a small, short-length, retrospective study (171 men and 166 women) intended to evaluate sex-related disparities in dyslipidemic patients treated with statins. The authors reported that women experienced a lower LDL-C decrease (−22.7 ± 11.8%) than men (−28.5 ± 11.8%), particularly in primary prevention, *p* < 0.001. In addition, the secondary outcome of the study was the assessment of lipid parameter variations in response to statins (atorvastatin 10 mg/dL, simvastatin 20 mg/dL, pravastatin 20 mg/dL, rosuvastatin 10 mg/dL, fluvastatin 80 mg/dL). A substantial reduction in total cholesterol (TC) and LDL-C levels was observed for atorvastatin and simvastatin in males vs. females (*p* < 0.05) [75] (Table 2). Specifically, atorvastatin and simvastatin are primarily metabolized by CYP3A4, which is expressed at a 2-fold greater level in women than in males, resulting in a quicker and more thorough statin metabolism and subsequently a decreased activity relative to men [76]. This discrepancy may explain the decreased LDL-C-reducing effects of these two medicines in the referred study. In the pravastatin and rosuvastatin groups, a greater decrease in TC and LDL-C levels was seen in males compared to females, although statistical significance was not attained. Another noteworthy finding was that in the simvastatin arm, males saw a substantial decline in HDL-C (−3.5 ± 2%), while women experienced a significant rise (6.1 ± 21.2%, *p* < 0.05 vs. men). Rosuvastatin induced a substantial upward trend in HDL-C levels in both sexes, with males having a higher rise than women (11.5 ± 12.3 vs. 2.5 ± 17.9%. *p* < 0.05). In primary prevention, women had a smaller LDL-C decrease than men, although the findings should be interpreted with caution, owing to the absence of randomization, small sample size, and short duration of follow-up [75] (Table 2). Sex equality in achieving the target lipid profile has been found in a more recent cohort study of 571 patients (289 women and 282 men) receiving atorvastatin or simvastatin for the first time. In contrast, the adjusted pairwise comparison indicated a significantly greater mean percentage increase in HDL-C levels in women than in men, after beginning statin treatment (*p* < 0.001) [77]. A newly published prospective observational study evaluated the attainment of LDL-C plasma levels of 1.8 mmol/L in 232 elderly patients (139 men and 93 women with an average age of 75.5 years) using statins (atorvastatin in dosages ranging from 10 to 80 mg and rosuvastatin 10–40 mg) for three months after acute coronary syndrome (ACS). While 56.5% of patients met the LDL-C target following 3 months of treatment, the percentage of women attaining their LDL-C target was significantly lower than that of men (40.9% vs. 66.6%, *p* < 0.001). Univariate logistic regression showed that females had a lower likelihood of achieving their LDL-C objective than males (OR 0.34, 95% CI 0.20–0.59). Of note, age, smoking, reduced physical activity, LDL-C levels on hospitalization, statin use history prior to admission, and high-intensity statin prescription at discharge exhibited a strong impact on LDL-C goal accomplishment [78] (Table 2). Cutting-edge developments in the area of genetics have shown that genetic polymorphisms affecting the activity of SLCO1B1 (a gene encoding the membrane anion transport polypeptide—OATP1B1—responsible for the aid of statin active uptake into hepatocytes from the blood) elevate simvastatin concentrations by 221% and atorvastatin concentrations by 144%, as a result of decreased absorption. Reduced hepatic concentration of statins diminishes the effectiveness of the LDL-C-lowering effect, while increased systemic exposure to statins enhances the risk of experiencing muscle weakness and pain. A 2022 study attempted to establish whether women taking simvastatin or atorvastatin were as likely as men to attain cholesterol levels below clinically high cut-off values. The study employed data from the UK Biobank, which included 69,185 of community volunteers (26,185 women, 41,445 men), who were tracked for almost 10 years in primary care and hospital electronic health records. Despite taking simvastatin or atorvastatin, female carriers of the SLCO1B1*5 genetic variation (n = 591, 2.24% of 26,185 females) were more likely to display elevated cholesterol plasma levels (48.8% vs. 41.7%, OR 1.31, 95% CI 1.1–1.55, *p* = 0.001). Men homozygous for the SLCO1B1*5 reduced function mutated gene (n = 927, 2.24% of 41,445 males) were likewise more susceptible to having elevated TC than SLCO1B1 regular function homozygotes (29.1% vs. 24.7%, OR 1.27, 95% CI 1.09–1.47, *p* = 0.001). The researchers suggest that SLCO1B1*5 genotype-guided statin selection may be required to increase the efficacy of statin treatment in women [79]. In 2004, Bennett et al. pioneered new approaches in research by establishing that the association of ezetimibe and statins yields major incremental decreases in LDL-C, as opposed to statin monotherapy. Ezetimibe belongs to the class of cholesterol absorption inhibitors, which impede the intestinal absorption of dietary and biliary cholesterol. Data from four RCTs were merged to investigate if ezetimibe combined with a statin is equally effective and safe in treating hypercholesterolemia in women and men (n = 1861, 1065 women, 796 men). Ezetimibe + statin indicated better efficacy in lowering serum concentrations of LDL-C, apolipoprotein B, and triglycerides (TGs) and elevating HDL-C compared to statin monotherapy. Similar positive outcomes of ezetimibe were seen in both males and women. The safety profile of participants who received ezetimibe + statin was comparable to that of patients who received statin alone, regardless of sex [80]. The pooled analysis conducted by Abramson et al. in 2011 provided the foundation for establishing that sex influences the clinical performance and the rate of adverse events of lipid-lowering agents (statins or statin + ezetimibe). Data from 27 RCTs (n = 22,231; 11,295 men, 10,499 women) revealed that an ezetimibe + statin mixture exhibited a substantially enhanced change in LDL-C (*p* = 0.0066), non-HDL-C, TC, TGs, HDL-C, apolipoprotein A-I (all *p* = 0.0001), and apolipoprotein B (*p* = 0.0055) in males compared to females. However, these variations were insignificant (2%), and their clinical significance is debatable. Regarding sex-specific adverse events, women experienced significantly higher rates of side effects, including gallbladder and gastrointestinal disorders and hypersensitivity and rash, for which women were more likely to discontinue the treatment. On the other hand, males reported considerably higher creatine kinase kinase elevations (up to 10 times the upper limit of normal) and hepatitis-related adverse events, which were much more prevalent in the combination group compared to the statin monotherapy cohort [81] (Table 2).

It is acknowledged that lipid-lowering drugs exhibit pleiotropic effects that modulate the release of bioactive peptides from adipose tissue. Sex influences the distribution of adipose tissue and the synthesis of adipokines [82,83]. The impaired synthesis of adipose tissue hormones such as leptin, adiponectin, visfatin, and tumor necrosis factor-α (TNF-*α*) is linked to the onset of atherosclerosis, type 2 diabetes, and insulin resistance [84,85]. Krysiak et al. were the first to investigate the influence of a 30-day treatment with atorvastatin and ezetimibe, administered individually or in combination, on adipose tissue hormone secretion in men and women with hypercholesterolemia. The sex-specific, retrospective assessment of 61 patients (26 women, 35 men) has concluded that the administration of simvastatin (40 mg per day) and ezetimibe (10 mg per day) taken alone or in combination did not influence the plasma adipokine levels. Irrespectively of sex, the administration of simvastatin and statin/ezetimibe combination decreased plasma levels of high-sensitivity C-reactive protein (hsCRP), free fatty acids (FFAs), leptin, visfatin, and TNF-*α* and raised plasma levels of adiponectin. Conversely, ezetimibe tended to lower plasma levels of hsCRP and had no effect on plasma levels of FFAs, leptin, visfatin, and TNF-*α*. To corroborate the results, research with a larger sample size and a longer duration of follow-up is warranted [86]. The same research team performed a different retrospective study stratified by sex, focusing on 69 individuals with type 2 diabetes and dyslipidemia who were treated with simvastatin (40 mg daily), fenofibrate (200 mg daily), or simvastatin + fenofibrate. The main purpose of the study was to identify if the reduction in proinflammatory cytokines following 12 weeks of treatment with hypolipidemic agents is sex-dependent. Intriguingly, when patients were treated only with simvastatin or fenofibrate, the monocyte-suppressing impact was similar for men and women. In contrast, when simvastatin and fenofibrate were used together, the modifications in monocyte release of interleukin-6 and MCP-1 caused by the medicines were more significant in men. In both sexes, the simvastatin/fenofibrate mixture lowered circulating levels of TC, LDL-C, and TGs more effectively than fenofibrate alone and was superior to simvastatin in influencing TC, HDL-C, TGs, glucose and HOMA-IR, and glycated hemoglobin [87].

Statins have long been prescribed for individuals with CVD, given that multiple clinical trials demonstrated their lipid-lowering benefits in ischemic heart disease (IHD) patients [88,89]. The latest research has shown that statins are connected with a decreased risk of dementia. A retrospective cohort study based on medical data gathered from 143,174 Korean patients with IHD aimed to explore the relationship between routinely administered statins (atorvastatin, simvastatin, rosuvastatin, pitavastatin, pravastatin, lovastatin, and fluvastatin) and the incidence of dementia in the elderly, taking into account sex, age, and exposure duration. Statin users displayed substantial protective benefits against the incidence of dementia in comparison to non-users, with an adjusted hazard ratio (HR) of 0.95, 95% CI 0.92–0.97. Of note, atorvastatin, rosuvastatin, pitavastatin, pravastatin, and fluvastatin were considered beneficial for lowering the dementia risk. In a subgroup analysis based on sex, the anti-dementia benefits of statin were confirmed, with an adjusted HR of 0.92, 95% CI 0.88–0.96, in men and an adjusted HR of 0.96, 95% CI 0.93–0.96, in women. Rosuvastatin and pravastatin considerably reduced HRs in both men and women. Nevertheless, atorvastatin was correlated with a decreased risk of dementia in men (adjusted HR 0.92, 95% CI, 0.88–0.97), but not in women (adjusted HR 0.97, 95% CI 0.94–1.00), and lovastatin was linked to a lower likelihood of dementia in women (adjusted HR 0.74, 95% CI 0.58–0.95), but not in men (adjusted HR 1.15, 95% CI 0.84–1.60). While the results might indicate that sex influences the association between statins and dementia, the findings should be carefully interpreted due to the risk of introducing confounding factors by the retrospective design of the study [90] (Table 2).

In order to address the noncardiac effects of simvastatin and pravastatin, a 2015 randomized double-blind sex-stratified study (324 women, 692 men) has provided compelling insights on the correlation between statins and the prevalence of aggressive behaviors [91]. Statins suppress the mevalonate pathway, thus interfering with the synthesis of the steroid hormone testosterone. In women without a history of aggressiveness, statins caused a substantial increase in hostility [92], and pravastatin, in particular, showed a stronger impact than simvastatin: regression coefficient 1.02, standard error (SE) = 0.43, *p* = 0.02. In the study population adjusted for the baseline Aggression Subscale of the Modified Overt Aggression Scale (OASMa), statin usage was linked with a tendency toward greater aggressiveness in women using statins. In contrast, there was a tendency toward a reduction in aggression and testosterone levels in men treated with simvastatin: regression coefficient −1.1, SE = 0.30, 95% CI = −1.7–−0.56, *p* = 0.0002. The scientists ascribed simvastatin’s activity not only to its well-known inhibitory effect on HMG-CoA reductase, but also to its influence on the final phases of testicular steroidogenesis: simvastatin blocks the 17-ketosteroid-oxidoreductase-mediated conversion of dehydroepiandrosterone and androstenedione to androstenediol [91].

An increasing number of studies indicate that lipid metabolism plays a crucial role in modulating leukocyte activity and global immunological responses, having an impact on autoimmune pathogenesis and antinuclear antibody status [93,94]. Animal studies have shown that cholesterol-rich atherogenic diets may cause or exacerbate autoimmune-like disorders [94,95]. Recent insights from a cross-sectional survey (n = 1526; 811 women and 715 men) shed light on the sex-specific link between serum lipid levels, statin administration, and antinuclear antibodies (ANAs)—typical clinical indicators of autoimmunity and immune disruption. Given the anti-inflammatory and protective benefits of statins in autoimmune disorders [96,97], the investigators found that women on statin therapy had considerably lower chances of being ANA+ (OR 0.25; 95% CI 0.09–0.76), but no significant correlation was detected between statin usage and ANA status in men. Irrespective of ANA status, the majority of men and women had adequate TC levels (<200 mg/dL). However, a higher percentage of ANA+ women than ANA+ men had elevated TC levels (>240 mg/dL) (13% vs. 9%). Moreover, independent of the ANA group, a higher proportion of women had low HDL-C levels. The research demonstrates that lipid metabolism is a potential target for preventing the development of autoimmune disease [71].

Increased triglyceride levels have been reported as a biomarker of cardiovascular risk in epidemiological and clinical trials. Mendelian randomization clinical studies demonstrate that TGs are involved in the pathogenesis of atherosclerotic disease. Fibrates, niacin, and omega-3 fatty acids are some of the triglyceride-lowering medications available on the pharmaceutical market [98]. A randomized clinical trial enrolling 5518 patients with type 2 diabetes (30.7% women) has reported astounding results concerning the use of fenofibrate in combination with statins in high-risk patients. There was a significant sex dependence favoring males (*p* = 0.01): females had a greater risk of CVD events with fenofibrate plus statin than with placebo plus statin (9.1% vs. 6.6%), but men had a reduced rate of CVD events with fenofibrate plus statin (11.2% vs. 13.3%). However, the research study showed that adding fenofibrate to statin therapy did not mitigate the incidence of CVD outcomes among diabetic individuals with increased CVD risk [99]. Another double-blind placebo-controlled trial has provided additional insights concerning the occurrence of cardiovascular events among patients with type 2 diabetes (n = 9795, 37.3% women). Women experienced significantly greater improvements with fenofibrate than men in terms of LDL-C (9.8% vs. 3.3%, *p* < 0.001) and TC (9.5% vs. 5.2%, *p* < 0.001), whereas changes in HDL-C and TG levels were comparable for both sexes. Additionally, there was no substantial improvement in the main composite outcome in the total population (HR 0.8; 95% CI 0.45–0.73; CI 0.75–1.05; *p* = 0.16). Total CVD occurrences were decreased by 20% in women (HR 0.8; 95 percent CI 0.64–0.99; *p* = 0.04) and by 8% in males (HR 0.92; 95 percent CI 0.81–1.05; *p* = 0.2) in subgroup analyses; however, the interaction analysis had a statistically insignificant *p*-value (*p* = 0.3) [100].

**Table 2:** Summary table of the publications that are part of this narrative review pertaining to dyslipidemia.

Study Reference	Study Design	Summary of Findings
Puri, R. et al. Sex-Related Differences of Coronary Atherosclerosis Regression Following Maximally Intensive Statin Therapy: Insights from Saturn (2014) [70]	randomized controlled trial	Rosuvastatin (40 mg) decreased overall atheroma volume regression in women compared to males
Karlson, B. W. et al. Effects of Age, Gender and Statin Dose on Lipid Levels: Results from the VOYAGER Meta-Analysis Database (2022) [73]	inception cohort study	In women, administration of rosuvastatin (5–10 mg), atorvastatin 10–80 mg, and simvastatin (10–80 mg) led to a greater reduction in LDL-C
Mombelli, G. et al. Gender-Related Lipid and/or Lipoprotein Responses to Statins in Subjects in Primary and Secondary Prevention (2015) [75]	retrospective observational study	In males, atorvastatin and simvastatin were associated with a significant reduction in total cholesterol (TC) and LDL-C levels. Rosuvastatin caused a significant increase in HDL-C in men compared to women
Nguyen, T. et al. Sex Difference in Control of Low-Density Lipoprotein Cholesterol in Older Patients after Acute Coronary Syndrome (2022) [78]	prospective observational study	Males had lower LDL-C levels after three months of atorvastatin (10–80 mg) and rosuvastatin (10–40 mg) following acute coronary syndrome than females
Abramson, B. L. et al. Response by Sex to Statin plus Ezetimibe or Statin Monotherapy: A Pooled Analysis of 22,231 Hyperlipidemic Patients (2011) [81]	pooled analysis of double-blind, active or placebo-controlled studies	Ezetimibe + statin caused gallbladder, gastrointestinal, hypersensitivity, and rash in women and creatine kinase increases and hepatitis in men
Kim, M. et al. Impact of Statin Use on Dementia Incidence in Elderly Men and Women with Ischemic Heart Disease (2020) [90]	retrospective cohort study	Atorvastatin lowers male dementia risk, whereas lovastatin lowers female dementia risk

### 2.3. Diabetes Mellitus

Type 2 diabetes mellitus (T2DM) is the sixth most frequent cause of death for men (3.2% of deaths) and the seventh for women (2.7%) [101]. T2DM occurs in young women at a higher rate than in men, while the incidence of type 2 diabetes rises with age in men [102]. These sex-specific variations can be identified during the prediabetic stage: women have a higher prevalence of impaired glucose tolerance, while men have a higher prevalence of impaired fasting blood glucose [103,104]. Moreover, studies have revealed sex disparities in the biological reactivity to antidiabetic drugs [105,106]. A sex- and body mass index (BMI)-stratified investigation has demonstrated the influence of sulfonylureas and thiazolidinediones (pioglitazone or rosiglitazone) on individuals receiving type 2 diabetes treatment. The findings have shown that in non-obese males, the average glycemic response to sulfonylureas is improved compared to that for thiazolidinediones, without additional weight gain, but with an elevated risk of hypoglycemia. On the other hand, the usage of thiazolidinedione tends to result in better glycemic regulation for obese women. However, women are considerably more likely to gain weight, develop edema, and suffer from bone fractures under thiazolidinedione treatment [107]. There is a clear difference in the response to antidiabetic drugs between women and men, as emphasized by the literature in the field. An excellent review by Franconi et al. addressing sex influences on the pharmacological response to various medicines has demonstrated that insulin has a different safety profile in men and women. To begin with, hypoglycemia occurs more often in women with T2DM, as they receive higher doses of insulin. Hypoglycemia is also responsible for the risk of falls and subsequently for the increased risk of bone fracture. Additionally, insulin therapy is often linked to an increased incidence of a variety of cancers, including sex-specific tumors such as breast cancer. Glargine in particular, a recombinant DNA analog of human insulin, raises the risk of breast malignancies. A woman’s propensity to develop breast cancer increases with her family’s history of the disease; for this, an assessment of the patient’s family background and personal medical history of the disease should be completed before starting glargine treatment. Moreover, metabolic changes in sex hormones that occur during the menstrual cycle can influence insulin demand, sensitivity, and glucose metabolism. It has been shown that fertile women in the second half of their menstrual cycle need a higher insulin dose [106]. Biguanide metformin is commonly used to treat T2DM since it reduces hepatic glucose synthesis and increases insulin sensitivity. A recently published narrative review puts the spotlight on sex disparities in the pharmacological response and side effects of metformin. The researchers hypothesized that women may receive lower dosages of metformin than males and suffer greater gastrointestinal side effects. It has been reported that the risk of cardiovascular events in women using metformin is lower than that in males. Due to the scarcity of more rigorous evidence from clinical investigations, clear conclusions could not be drawn [108]. According to a longitudinal survey of 1712 patients with T2DM (1011 men, 701 women), women are substantially more likely to experience adverse effects (nausea, stomach pain, flatulence, etc.) than males, following 2 weeks (34% versus 25%, *p* = 0.001) and 6 weeks (34% versus 28%, *p* = 0.001) of treatment with metformin. Nevertheless, after a year of metformin therapy, the rate of adverse events decreased in a manner comparable to that of males [109] (Table 3). A recent study employing data from a Chinese RCT trial (640 participants, 392 men, 248 women) found that after 24 and 48 weeks of metformin therapy, women achieved lower fasting glucose levels and two-hour postprandial blood sugar than men. Interestingly, at both 24 and 48 weeks, the drop in two-hour postprandial glucose in men treated with acarbose was considerably larger than in those treated with metformin. Moreover, female subjects treated with metformin exhibited an enhancement in insulin secretion, while male patients exhibited no considerable improvement [110] (Table 3).

Metformin has been linked to a lower incidence of breast cancer and colorectal cancer (CRC) in women and hepatocellular carcinoma in men [105]. A meta-analysis revealed that CRC patients with T2DM had a 17% higher risk of total mortality. In recent years, accumulating data have shown that metformin also inhibits cancer. A 2020 meta-analysis comprising eight cohort studies reported that metformin decreased the overall mortality of CRC patients with T2DM (HR = 0.80, 95% CI 0.67–0.95), with women exhibiting a lower CRC-specific death rate than men (HR = 0.63, 95% CI = 0.41–0.97) [111].

CVD is the main cause of death and morbidity among individuals with T2DM, and females are twice as likely as males to develop it. Randomized, controlled studies established that newer glucose-lowering medications are cardioprotective, although the majority of the subjects were males. A 2020 observational study including 167,254 diabetes patients (90,674 men, 76,580 women) investigated the consequences of switching from metformin therapy to newer antidiabetics, such as sodium-glucose-like transport-2 inhibitors (SGLT-2is), glucagon-like peptide-1 receptor agonists (GLP-1RAs), and dipeptidyl peptidase-4 inhibitors (DDP-4s). The results showed that the risk of cardiovascular events was reduced in women compared to men, after a median observation span of 4.5 years (14.7 vs. 16.7). Compared to sulfonylureas, the combined use of more modern glucose-lowering medications with metformin was linked with a decreased incidence of serious cardiovascular adverse events: GLP-1RAs (adjusted HR for women: 0.57, 95% CI: 0.48–0.68; adjusted HR for men: 0.82, 0.71–0.95), dipeptidyl peptidase-4 inhibitors (adjusted HR for women: 0.83, 0.77–0.89; adjusted HR for men: 0.85, 0.79–0.91), and SGLT-2is (adjusted HR for women: 0.58, 0.46–0.74; adjusted HR for men: 0.69, 0.57–0.83). This positive impact was more significant in females than in males, particularly among users of GLP-1RAs (*p* = 0.002). The drugs showed an overall good safety, with SGLT-2is having a superior safety profile compared to GLP-1RAs, regardless of sex (adjusted HR: 0.84, 95% CI: 0.75–0.95) [112] (Table 3). Wang et al. combined data from spontaneous reporting systems and digital health records to assess sex variations in myocardial infarction related to oral antidiabetic drugs such as metformin, sulfonylureas, alpha-glucosidase inhibitors (AGIs), thiazolidinediones, meglitinides, and DPP-4. Men had a greater risk of metformin-related (OR 1.14; 99% CI 1.03–1.26) and sulfonylurea-related myocardial infarction than women (OR 1.13; 99% CI 1.02–1.25). In contrast, female pioglitazone users had a greater risk of myocardial infarction than men (OR of the multiplicative interaction 0.76, 95% CI: 0.59–0.98). The findings clearly demonstrate that sex–drug interactions are a fundamental aspect of establishing a therapeutic approach for T2DM [113] (Table 3). A small trial of 41 diabetic patients (20 men and 21 women) revealed that DPP-4 therapy with gliptin for 6 months resulted in exclusive benefits for women: a substantial drop in body weight (85.34 ± 17.7 kg to 83.32 ± 17.55 kg, *p* = 0.02) and a lowering effect on hepatic and myocardial lipid concentrations (*p* = 0.03 for hepatic fat content reduction, *p* = 0.01 for myocardial fat content reduction) [114]. GLP-1RAs are antidiabetic medicines that imitate the activity of natural glucagon-like peptide 1. In addition to producing a potent hypoglycemic effect, they also have cardiorenal-protective and neuroprotective properties, are antimicrobial, stimulate fat loss, are appetite suppressants, and have a favorable safety profile, especially with regard to the risk of hypoglycemia [115].

A narrative review published in 2022 concluded that male and female responses to GLP-1RAs varied to some degree in terms of weight reduction and the prevalence of gastrointestinal side effects (in women). Regarding other parameters, namely HbA1c levels, hypoglycemia risk, and cardiovascular risk, it remains to be seen whether there is a sex difference. Addressing other indicators, such as hemoglobin A1c (HbA1c) levels, hypoglycemia risk, and cardiovascular risk, no sex differences were observed. The authors highlighted that the retrieved clinical data featured heterogeneous patient groups and, therefore, a high bias tendency [116].

The visceral adipocytes cells secrete Omentin-1, a unique adipokine with insulin-sensitizing properties [117]. Moreover, studies have shown a correlation between low omentin levels and insulin-resistant conditions such as obesity, diabetes, and polycystic ovarian syndrome [118,119,120]. Leptin, the 167 amino acid product of the human gene for obesity, is involved in glucose and insulin signaling pathways through various mechanisms [121]. It is hypothesized that a rise in plasma leptin inhibits insulin secretion from pancreatic *β*-cells, hence promoting peripheral insulin sensitivity [121]. A small randomized clinical trial included 91 newly diagnosed T2DM patients who randomly received daily doses of 1000 mg metformin or 30 mg pioglitazone. The primary objective of the research was to evaluate the sex-specific effects of metformin and pioglitazone monotherapy on serum levels of omentin and leptin. Researchers concluded that metformin and pioglitazone were similarly efficacious in lowering omentin and leptin levels.

Regarding sex dissimilarities after three months of treatment, metformin lowered the levels of omentin and leptin in women, but just leptin in males. Pioglitazone, in contrast, lowered both adipokines in women, while no changes were observed in males. Nonetheless, the results should be interpreted with caution, owing to the small sample size and brief follow-up duration [122].

**Table 3:** Summary table of the publications that are part of this narrative review pertaining to diabetes mellitus.

Study Reference	Study Design	Summary of Findings
de Vries, S. T. et al. Sex Differences in Adverse Drug Reactions of Metformin: A Longitudinal Survey Study (2020) [109]	longitudinal survey study	Women are more prone than men to develop metformin-related side effects such as nausea, stomach discomfort, and flatulence
Li, J. et al. Gender-Differential Effects on Blood Glucose Levels between Acarbose and Metformin in Chinese Patients with Newly Diagnosed Type 2 Diabetes: A Sub-Analysis of the March Trial (2021) [110]	randomized controlled, open-label, multicenter trial	Men treated with acarbose had a significant decline in two-hour postprandial glucose, whereas women treated with metformin had lower fasting glucose and two-hour postprandial glucose
Raparelli, V. et al. Sex Differences in Cardiovascular Effectiveness of Newer Glucose-Lowering Drugs Added to Metformin in Type 2 Diabetes Mellitus (2020) [112]	population-based analysis of randomized controlled trials	Sodium-glucose-like transport-2 inhibitors (SGLT-2is), glucagon-like peptide-1 receptor agonists (GLP-1RAs), and dipeptidyl peptidase-4 inhibitors (DDP-4s) lessen women’s cardiovascular risk relative to males
Wang, S. H. et al. Use of Spontaneous Reporting Systems to Detect Host-Medication Interactions: Sex Differences in Oral Anti-Diabetic Drug-Associated Myocardial Infarction (2018) [113]	registry analysis	Men had a higher risk of heart attack with metformin and sulfonylureas than women. Female pioglitazone users had a higher risk of myocardial infarction than men

## 3. Materials and Methods

The first step in the creation of the present review was the selection of the topic. Three main diseases were chosen based on their incidence and importance in everyday practice: COVID-19, diabetes mellitus, and dyslipidemia. The research was conducted on 2 electronic databases (PubMed and Google Scholar) from 1 March 2022 until 1 January 2023. The search terms used in different combinations consisted of a first word representing the disease or the pharmacological class/substance (i.e., SARS-CoV-2, COVID-19, chloroquine, hydroxychloroquine, remdesivir, favipiravir, lopinavir-ritonavir, tocilizumab, plasma, immunoglobulins, statin, lipid-lowering medication, HMG-CoA reductase inhibitors, fibrate, niacinamide, nicotinic acid, bile acid sequestrant, cholesterol absorption inhibitor, dyslipidemia, hyperlipidemia, hypertriglyceridemia, Sulfonylureas, meglitinides, metformin, thiazolidinediones, alpha-glucosidase inhibitors, dipeptidyl peptidase IV inhibitors, Sodium-glucose transport protein 2 inhibitors, glucagon-like peptide 1 receptor agonists, hypoglycemic drugs) AND a second word representing the main focus on sex/gender variations (i.e., gender dissimilarities, sex differences, sex-stratified).

A total of 1879 records were identified through primary screening conducted by clinical pharmacist interns (A.L., M.R., N.J.). The records were further evaluated, and duplicates were removed (n = 324). Pertinent review articles were also screened for supplementary references that might have been missed in the primary search. Based on their title and abstract, the records were disqualified from consideration if they fit into one of the following categories: non-original research studies, conference abstracts, editorials, letters to the editor, non-human studies, or manuscripts published in a language other than English. A second screening of the remaining publications was performed during in-person meetings of a group of specialists (a cardiologist, an internal physician, a family physician and pharmacologist, and a clinical pharmacist). The group excluded articles that failed to successfully identify distinct pharmacological responses in men and women, provided insufficient data, or presented methodological flaws. In order to reach a consensus and reduce the possibility of bias and misinterpretation, all members (including clinical pharmacist interns) participated in a follow-up meeting that addressed data validation and solved any major disagreements. Finally, a number of 93 eligible articles were scrutinized end-to-end and were included in the present review.

## 4. Limitations

This review was not exempt from limitations. The present narrative review has centered on the dichotomous constructs of male and female, men and women. However, it is acknowledged that the complexities of sex and gender in the medical domain encompass a spectrum, which includes transgender, intersex, and non-binary identities, among other examples of diversity that accurately mirror the broader population. Furthermore, a significant proportion of the studies incorporated in this review had sample sizes that were below 100. Non-randomized and observational studies possess inherent limitations and biases. Several studies did not report the standard deviation. Additionally, solely research papers that were published in the English language were incorporated.

## 5. Conclusions

This narrative review highlights the importance of considering sex differences in pharmacological response in the context of SARS-CoV-2 infection, dyslipidemia, and diabetes mellitus. The evidence suggests that there are significant sex-related differences in disease severity, response to treatment, and risk of complications. These differences may be influenced by various factors, including immunological responses, genetics, and hormones. The present study has identified certain shortcomings in the existing literature pertaining to the inadequate utilization of sex and gender in the context of clinical research. Incorporating sex-based considerations in clinical practice has the potential to customize medical care for individuals by taking into account fundamental biological distinctions between males and females. Future research should include more trials of a higher caliber. In order to determine if sex is an impact modifier rather than a confounding variable, researchers should think about how sex is employed in studies, preferring mediation analyses. This can subsequently contribute to enhanced health outcomes and patient experiences and lessened disparities in care.

## References

[1] Giacobbe G., Granata V., Trovato P., Fusco R., Simonetti I., De Muzio F., Cutolo C., Palumbo P., Borgheresi A., Flammia F. (2023). Gender Medicine in Clinical Radiology Practice. J. Pers. Med..

[2] Nachtigall I., Bonsignore M., Hohenstein S., Bollmann A., Günther R., Kodde C., Englisch M., Ahmad-Nejad P., Schröder A., Glenz C. (2022). Effect of Gender, Age and Vaccine on Reactogenicity and Incapacity to Work after COVID-19 Vaccination: A Survey among Health Care Workers. BMC Infect. Dis..

[3] Tamargo J., Rosano G., Walther T., Duarte J., Niessner A., Kaski J.C., Ceconi C., Drexel H., Kjeldsen K., Savarese G. (2017). Gender Differences in the Effects of Cardiovascular Drugs. Eur. Hear. J.-Cardiovasc. Pharmacother..

[4] Romanescu M., Buda V., Lombrea A., Andor M., Ledeti I., Suciu M., Danciu C., Dehelean C.A., Dehelean L. (2022). Sex-Related Differences in Pharmacological Response to CNS Drugs: A Narrative Review. J. Pers. Med..

[5] Farkouh A., Riedl T., Gottardi R., Czejka M., Kautzky-Willer A. (2020). Sex-Related Differences in Pharmacokinetics and Pharmacodynamics of Frequently Prescribed Drugs: A Review of the Literature. Adv. Ther..

[6] Moores G., Wolff E., Pikula A., Bui E. (2022). Sex Differences in Neurology: A Scoping Review Protocol. BMJ Open.

[7] (2016). H2020 Programme Guidance on Gender Equality in Horizon 2020. History of Changes. Guidance on Gender Equality in Horizon 2020. https://eige.europa.eu/sites/default/files/h2020-hi-guide-gender_en.pdf.

[8] CIHR What Is Gender? What Is Sex?. https://cihr-irsc.gc.ca/e/48642.html.

[9] Nabil Abdelnaim Mohamed F. (2021). The Ethicist’s Practical Guide to the Evaluation of Preclinical Research from a Sex and Gender Perspective.

[10] Dickenson D. (2006). Gender and Ethics Committees: Where’s the ‘Different Voice’?. Bioethics.

[11] Saxena A., Lasher E., Somerville C., Heidari S. (2022). Considerations of Sex and Gender Dimensions by Research Ethics Committees: A Scoping Review. Int. Health.

[12] Mittelstrass K., Ried J.S., Yu Z., Krumsiek J., Gieger C., Prehn C., Roemisch-Margl W., Polonikov A., Peters A., Theis F.J. (2011). Discovery of Sexual Dimorphisms in Metabolic and Genetic Biomarkers. PLoS Genet..

[13] Parks B.W., Sallam T., Mehrabian M., Psychogios N., Hui S.T., Norheim F., Castellani L.W., Rau C.D., Pan C., Phun J. (2015). Genetic Architecture of Insulin Resistance in the Mouse. Cell Metab..

[14] White U.A., Tchoukalova Y.D. (2014). Sex Dimorphism and Depot Differences in Adipose Tissue Function. Biochim. Biophys. Acta (BBA)-Mol. Basis Dis..

[15] Karp N.A., Mason J., Beaudet A.L., Benjamini Y., Bower L., Braun R.E., Brown S.D.M., Chesler E.J., Dickinson M.E., Flenniken A.M. (2017). Prevalence of Sexual Dimorphism in Mammalian Phenotypic Traits. Nat. Commun..

[16] Kukurba K.R., Parsana P., Balliu B., Smith K.S., Zappala Z., Knowles D.A., Favé M.-J., Davis J.R., Li X., Zhu X. (2016). Impact of the X Chromosome and Sex on Regulatory Variation. Genome Res..

[17] Arnold A.P., Lusis A.J. (2012). Understanding the Sexome: Measuring and Reporting Sex Differences in Gene Systems. Endocrinology.

[18] Krishnan K.C., Mehrabian M., Lusis A.J. (2018). Sex Differences in Metabolism and Cardiometabolic Disorders. Curr. Opin. Lipidol..

[19] Madla C.M., Gavins F.K.H., Merchant H.A., Orlu M., Murdan S., Basit A.W. (2021). Let’s Talk about Sex: Differences in Drug Therapy in Males and Females. Adv. Drug Deliv. Rev..

[20] Donovan M.D. (2005). Sex and Racial Differences in Pharmacological Response: Effect of Route of Administration and Drug Delivery System on Pharmacokinetics. J. Women’s Health.

[21] Dressman J.B., Berardi R.R., Dermentzoglou L.C., Russell T.L., Schmaltz S.P., Barnett J.L., Jarvenpaa K.M. (1990). Upper Gastrointestinal (GI) PH in Young, Healthy Men and Women. Pharm. Res..

[22] Sadik R., Abrahamsson H., Stotzer P.-O. (2003). Gender Differences in Gut Transit Shown with a Newly Developed Radiological Procedure. Scand. J. Gastroenterol..

[23] Gandhi M., Aweeka F., Greenblatt R.M., Blaschke T.F. (2004). Sex Differences in Pharmacokinetics and Pharmacodynamics. Annu. Rev. Pharmacol. Toxicol..

[24] Soldin O.P., Mattison D.R. (2009). Sex Differences in Pharmacokinetics and Pharmacodynamics. Clin. Pharmacokinet..

[25] Klein S.L., Huber S. (2010). Sex Differences in Susceptibility to Viral Infection. Sex Hormones and Immunity to Infection.

[26] Klein S.L., Jedlicka A., Pekosz A. (2010). The Xs and Y of Immune Responses to Viral Vaccines. Lancet Infect. Dis..

[27] Hewagama A., Patel D., Yarlagadda S., Strickland F.M., Richardson B.C. (2009). Stronger Inflammatory/Cytotoxic T-Cell Response in Women Identified by Microarray Analysis. Genes Immun..

[28] Grasselli G., Zangrillo A., Zanella A., Antonelli M., Cabrini L., Castelli A., Cereda D., Coluccello A., Foti G., Fumagalli R. (2020). Baseline Characteristics and Outcomes of 1591 Patients Infected with SARS-CoV-2 Admitted to ICUs of the Lombardy Region, Italy. JAMA.

[29] Palaiodimos L., Kokkinidis D.G., Li W., Karamanis D., Ognibene J., Arora S., Southern W.N., Mantzoros C.S. (2020). Severe Obesity, Increasing Age and Male Sex Are Independently Associated with Worse in-Hospital Outcomes, and Higher in-Hospital Mortality, in a Cohort of Patients with COVID-19 in the Bronx, New York. Metabolism.

[30] Stopsack K.H., Mucci L.A., Antonarakis E.S., Nelson P.S., Kantoff P.W. (2020). TMPRSS2 and COVID-19: Serendipity or Opportunity for Intervention?. Cancer Discov..

[31] Maleki Dana P., Sadoughi F., Hallajzadeh J., Asemi Z., Mansournia M.A., Yousefi B., Momen-Heravi M. (2020). An Insight into the Sex Differences in COVID-19 Patients: What Are the Possible Causes?. Prehosp. Disaster Med..

[32] Mohamed M.S., Moulin T.C., Schiöth H.B. (2020). Sex Differences in COVID-19: The Role of Androgens in Disease Severity and Progression. Endocrine.

[33] Goren A., Wambier C.G., Herrera S., McCoy J., Vaño-Galván S., Gioia F., Comeche B., Ron R., Serrano-Villar S., Ramos P.M. (2020). Anti-Androgens May Protect against Severe COVID-19 Outcomes: Results from a Prospective Cohort Study of 77 Hospitalized Men. J. Eur. Acad. Dermatol. Venereol..

[34] McCoy J., Cadegiani F.A., Wambier C.G., Herrera S., Vaño-Galván S., Mesinkovska N.A., Ramos P.M., Shapiro J., Sinclair R., Tosti A. (2020). 5-Alpha-Reductase Inhibitors Are Associated with Reduced Frequency of COVID-19 Symptoms in Males with Androgenetic Alopecia. J. Eur. Acad. Dermatol. Venereol..

[35] Calderone A., Menichetti F., Santini F., Colangelo L., Lucenteforte E., Calderone V. (2020). Selective Estrogen Receptor Modulators in COVID-19: A Possible Therapeutic Option?. Front. Pharmacol..

[36] Khan S., Ali A., Shi H., Siddique R., Shabana, Nabi G., Hu J., Wang T., Dong M., Zaman W. (2020). COVID-19: Clinical Aspects and Therapeutics Responses. Saudi Pharm. J..

[37] Estrogen Patch for COVID-19 Symptoms—Full Text View—ClinicalTrials.gov. https://clinicaltrials.gov/ct2/show/NCT04359329.

[38] Schiffer V.M.M.M., Janssen E.B.N.J., van Bussel B.C.T., Jorissen L.L.M., Tas J., Sels J.-W.E.M., Bergmans D.C.J., Dinh T.H.T., van Kuijk S.M.J., Hana A. (2020). The “Sex Gap” in COVID-19 Trials: A Scoping Review. EClinicalMedicine.

[39] Beigel J.H., Tomashek K.M., Dodd L.E., Mehta A.K., Zingman B.S., Kalil A.C., Hohmann E., Chu H.Y., Luetkemeyer A., Kline S. (2020). Remdesivir for the Treatment of COVID-19—Final Report. N. Engl. J. Med..

[40] Caruso C., Marcon G., Accardi G., Aiello A., Calabrò A., Ligotti M.E., Tettamanti M., Franceschi C., Candore G. (2023). Role of Sex and Age in Fatal Outcomes of COVID-19: Women and Older Centenarians Are More Resilient. Int. J. Mol. Sci..

[41] Sakoulas G., Geriak M., Kullar R., Greenwood K.L., Habib M., Vyas A., Ghafourian M., Dintyala V.N.K., Haddad F. (2020). Intravenous Immunoglobulin plus Methylprednisolone Mitigate Respiratory Morbidity in Coronavirus Disease 2019. Crit. Care Explor..

[42] Zeng F., Dai C., Cai P., Wang J., Xu L., Li J., Hu G., Wang Z., Zheng F., Wang L. (2020). A Comparison Study of SARS-CoV-2 IgG Antibody between Male and Female COVID-19 Patients: A Possible Reason Underlying Different Outcome between Sex. J. Med. Virol..

[43] Stiehm E.R. (2013). Adverse Effects of Human Immunoglobulin Therapy. Transfus. Med. Rev..

[44] Mirra D., Esposito R., Spaziano G., Rafaniello C., Iovino P., Cione E., Gallelli L., D’Agostino B. (2023). Association between Sex-Related *ALOX5* Gene Polymorphisms and Lung Atopy Risk. J. Clin. Med..

[45] Quesada-Gomez J.M., Entrenas-Castillo M., Bouillon R. (2020). Vitamin D Receptor Stimulation to Reduce Acute Respiratory Distress Syndrome (ARDS) in Patients with Coronavirus SARS-CoV-2 Infections: Revised Ms SBMB 2020_166. J. Steroid Biochem. Mol. Biol..

[46] Sanders J.M., Monogue M.L., Jodlowski T.Z., Cutrell J.B. (2020). Pharmacologic Treatments for Coronavirus Disease 2019 (COVID-19): A Review. JAMA.

[47] Gallelli L., Mannino G.C., Luciani F., de Sire A., Mancuso E., Gangemi P., Cosco L., Monea G., Averta C., Minchella P. (2021). Vitamin d Serum Levels in Subjects Tested for SARS-CoV-2: What Are the Differences among Acute, Healed, and Negative COVID-19 Patients? A Multicenter Real-Practice Study. Nutrients.

[48] Kalinke U., Barouch D.H., Rizzi R., Lagkadinou E., Türeci Ö., Pather S., Neels P. (2022). Clinical Development and Approval of COVID-19 Vaccines. Expert Rev. Vaccines.

[49] Flanagan K.L., Fink A.L., Plebanski M., Klein S.L. (2017). Sex and Gender Differences in the Outcomes of Vaccination over the Life Course. Annu. Rev. Cell Dev. Biol..

[50] Spini A., Giudice V., Brancaleone V., Morgese M.G., De Francia S., Filippelli A., Ruggieri A., Ziche M., Ortona E., Cignarella A. (2021). Sex-Tailored Pharmacology and COVID-19: Next Steps towards Appropriateness and Health Equity. Pharmacol. Res..

[51] Baden L.R., El Sahly H.M., Essink B., Kotloff K., Frey S., Novak R., Diemert D., Spector S.A., Rouphael N., Creech C.B. (2020). Efficacy and Safety of the MRNA-1273 SARS-CoV-2 Vaccine. N. Engl. J. Med..

[52] Polack F.P., Thomas S.J., Kitchin N., Absalon J., Gurtman A., Lockhart S., Perez J.L., Marc G.P., Moreira E.D., Zerbini C. (2020). Safety and Efficacy of the BNT162b2 MRNA COVID-19 Vaccine. N. Engl. J. Med..

[53] Logunov D.Y., Dolzhikova I.V., Shcheblyakov D.V., Tukhvatulin A.I., Zubkova O.V., Dzharullaeva A.S., Kovyrshina A.V., Lubenets N.L., Grousova D.M., Erokhova A.S. (2021). Safety and Efficacy of an RAd26 and RAd5 Vector-Based Heterologous Prime-Boost COVID-19 Vaccine: An Interim Analysis of a Randomised Controlled Phase 3 Trial in Russia. Lancet.

[54] Ewer K.J., Barrett J.R., Belij-Rammerstorfer S., Sharpe H., Makinson R., Morter R., Flaxman A., Wright D., Bellamy D., Bittaye M. (2021). T Cell and Antibody Responses Induced by a Single Dose of ChAdOx1 NCoV-19 (AZD1222) Vaccine in a Phase 1/2 Clinical Trial. Nat. Med..

[55] Gee J., Marquez P., Su J., Calvert G.M., Liu R., Myers T., Nair N., Martin S., Clark T., Markowitz L. (2021). First Month of COVID-19 Vaccine Safety Monitoring—United States, December 14, 2020–January 13, 2021. Morb. Mortal. Wkly. Rep..

[56] Tobaiqy M., Elkout H., MacLure K. (2021). Analysis of Thrombotic Adverse Reactions of COVID-19 AstraZeneca Vaccine Reported to EudraVigilance Database. Vaccines.

[57] Heidari S., Palmer-Ross A., Goodman T. (2021). A Systematic Review of the Sex and Gender Reporting in COVID-19 Clinical Trials. Vaccines.

[58] McMahon D.E., Amerson E., Rosenbach M., Lipoff J.B., Moustafa D., Tyagi A., Desai S.R., French L.E., Lim H.W., Thiers B.H. (2021). Cutaneous Reactions Reported after Moderna and Pfizer COVID-19 Vaccination: A Registry-Based Study of 414 Cases. J. Am. Acad. Dermatol..

[59] Shimabukuro T. (2021). Allergic Reactions Including Anaphylaxis after Receipt of the First Dose of Pfizer-BioNTech COVID-19 Vaccine—United States, December 14–23, 2020. Am. J. Transplant..

[60] Ma Y., Zhang Y., Li S., Yang H., Li H., Cao Z., Xu F., Sun L., Wang Y. (2021). Sex Differences in Association between Anti-Hypertensive Medications and Risk of COVID-19 in Middle-Aged and Older Adults. Drugs Aging.

[61] Ference B.A., Ginsberg H.N., Graham I., Ray K.K., Packard C.J., Bruckert E., Hegele R.A., Krauss R.M., Raal F.J., Schunkert H. (2017). Low-Density Lipoproteins Cause Atherosclerotic Cardiovascular Disease. 1. Evidence from Genetic, Epidemiologic, and Clinical Studies. A Consensus Statement from the European Atherosclerosis Society Consensus Panel. Eur. Heart J..

[62] Mach F., Baigent C., Catapano A.L., Koskinas K.C., Casula M., Badimon L., Chapman M.J., De Backer G.G., Delgado V., Ference B.A. (2020). 2019 ESC/EAS Guidelines for the Management of Dyslipidaemias: Lipid Modification to Reduce Cardiovascular Risk: The Task Force for the Management of Dyslipidaemias of the European Society of Cardiology (ESC) and European Atherosclerosis Society (EAS). Eur. Heart J..

[63] Cangemi R., Loffredo L., Carnevale R., Perri L., Patrizi M.P., Sanguigni V., Pignatelli P., Violi F. (2008). Early Decrease of Oxidative Stress by Atorvastatin in Hypercholesterolaemic Patients: Effect on Circulating Vitamin E. Eur. Heart J..

[64] Gelosa P., Cimino M., Pignieri A., Tremoli E., Guerrini U., Sironi L. (2007). The Role of HMG-CoA Reductase Inhibition in Endothelial Dysfunction and Inflammation. Vasc. Health Risk Manag..

[65] Spence J.D., Pilote L. (2015). Importance of Sex and Gender in Atherosclerosis and Cardiovascular Disease. Atherosclerosis.

[66] Wang X., Magkos F., Mittendorfer B. (2011). Sex Differences in Lipid and Lipoprotein Metabolism: It’s Not Just about Sex Hormones. J. Clin. Endocrinol. Metab..

[67] Matthan N.R., Jalbert S.M., Barrett P.H.R., Dolnikowski G.G., Schaefer E.J., Lichtenstein A.H. (2008). Gender-Specific Differences in the Kinetics of Nonfasting TRL, IDL, and LDL Apolipoprotein B-100 in Men and Premenopausal Women. Arterioscler. Thromb. Vasc. Biol..

[68] Schaefer E.J., Zech L.A., Jenkins L.L., Bronzert T.J., Rubalcaba E.A., Lindgren F.T., Aamodt R.L., Brewer H.B.J. (1982). Human Apolipoprotein A-I and A-II Metabolism. J. Lipid Res..

[69] Manteuffel M., Williams S., Chen W., Verbrugge R.R., Pittman D.G., Steinkellner A. (2014). Influence of Patient Sex and Gender on Medication Use, Adherence, and Prescribing Alignment with Guidelines. J. Women’s Health.

[70] Puri R., Nissen S.E., Shao M., Ballantyne C.M., Barter P.J., Chapman M.J., Erbel R., Libby P., Raichlen J.S., Uno K. (2014). Sex-Related Differences of Coronary Atherosclerosis Regression Following Maximally Intensive Statin Therapy: Insights from Saturn. JACC Cardiovasc. Imaging.

[71] Andersen C.J., Vance T.M. (2022). Sex-Specific Associations between Serum Lipids, Antinuclear Antibodies, and Statin Use in National Health and Nutrition Examination Surveys 1999–2004. Front. Med..

[72] Al-Zakwani I., Al-Mahruqi F., Al-Rasadi K., Shehab A., Al Mahmeed W., Arafah M., Al-Hinai A.T., Al Tamimi O., Al Awadhi M., Santos R.D. (2018). Sex Disparity in the Management and Outcomes of Dyslipidemia of Diabetic Patients in the Arabian Gulf: Findings from the CEPHEUS Study. Lipids Health Dis..

[73] Karlson B.W., Palmer M.K., Nicholls S.J., Barter P.J., Lundman P. (2017). Effects of Age, Gender and Statin Dose on Lipid Levels: Results from the VOYAGER Meta-Analysis Database. Atherosclerosis.

[74] Fulcher J., O’Connell R., Voysey M., Emberson J., Blackwell L., Mihaylova B., Simes J., Collins R., Kirby A., Colhoun H. (2015). Efficacy and Safety of LDL-Lowering Therapy among Men and Women: Meta-Analysis of Individual Data from 174000 Participants in 27 Randomised Trials. Lancet.

[75] Mombelli G., Bosisio R., Calabresi L., Magni P., Pavanello C., Pazzucconi F., Sirtori C.R. (2015). Gender-Related Lipid and/or Lipoprotein Responses to Statins in Subjects in Primary and Secondary Prevention. J. Clin. Lipidol..

[76] Wolbold R., Klein K., Burk O., Nüssler A.K., Neuhaus P., Eichelbaum M., Schwab M., Zanger U.M. (2003). Sex Is a Major Determinant of CYP3A4 Expression in Human Liver. Hepatology.

[77] Hunt N.B., Emmens J.E., Irawati S., de Vos S., Bos J.H.J., Wilffert B., Hak E., de Boer R.A. (2022). Sex Disparities in the Effect of Statins on Lipid Parameters The PharmLines Initiative. Medicine.

[78] Nguyen T.V., Tran D.T.T., Ngo T.T.K., Nguyen T.N. (2022). Sex Difference in Control of Low-Density Lipoprotein Cholesterol in Older Patients after Acute Coronary Syndrome. Geriatrics.

[79] Türkmen D., Masoli J.A.H., Kuo C.L., Bowden J., Melzer D., Pilling L.C. (2022). Statin Treatment Effectiveness and the SLCO1B1*5 Reduced Function Genotype: Long-Term Outcomes in Women and Men. Br. J. Clin. Pharmacol..

[80] Bennett S., Sager P., Lipka L., Melani L., Suresh R., Veltri E. (2004). Consistency in Efficacy and Safety of Ezetimibe Coadministered with Statins for Treatment of Hypercholesterolemia in Women and Men. J. Women’s Health.

[81] Abramson B.L., Benlian P., Hanson M.E., Lin J., Shah A., Tershakovec A.M. (2011). Response by Sex to Statin plus Ezetimibe or Statin Monotherapy: A Pooled Analysis of 22,231 Hyperlipidemic Patients. Lipids Health Dis..

[82] Govindarajan G., Alpert M.A., Tejwani L. (2008). Endocrine and Metabolic Effects of Fat: Cardiovascular Implications. Am. J. Med..

[83] Ahima R.S., Osei S.Y. (2008). Adipokines in Obesity. Obes. Metab..

[84] Hajer G.R., Van Haeften T.W., Visseren F.L.J. (2008). Adipose Tissue Dysfunction in Obesity, Diabetes, and Vascular Diseases. Eur. Heart J..

[85] Bienek R., Marek B., Kajdaniuk D., Borgiel-Marek H., Piecha T., Nowak M., Siemińska L., Głogowska-Szeląg J., Foltyn W., Kos-Kudła B. (2012). Adiponectin, Leptin, Resistin and Insulin Blood Concentrations in Patients with Ischaemic Cerebral Stroke. Endokrynol. Pol..

[86] Krysiak R., Zmuda W., Marek B., Okopień B. (2015). Comparison of the Effects of Short-Term Hypolipidaemic Treatment on Plasma Adipokine Levels in Men and Women with Isolated Hypercholesterolaemia. Endokrynol. Pol..

[87] Krysiak R., Gdula-Dymek A., Marek B., Okopień B. (2015). Comparison of the Effects of Hypolipidaemic Treatment on Monocyte Proinflammatory Cytokine Release in Men and Women with Type 2 Diabetes and Atherogenic Dyslipidaemia. Endokrynol. Pol..

[88] Kuwabara M., Kondo F., Hamada T., Takahashi J., Takenaka N., Furuno T. (2016). Impact of Statins Therapy for Ischemic Heart Disease Patients with Low-Density Lipoprotein Cholesterol Levels Less than 100 Mg/DL. Acta Cardiol. Sin..

[89] Shepherd J., Blauw G.J., Murphy M.B., Bollen E.L.E.M., Buckley B.M., Cobbe S.M., Ford I., Gaw A., Hyland M., Jukema J.W. (2002). Pravastatin in Elderly Individuals at Risk of Vascular Disease (PROSPER): A Randomised Controlled Trial. Lancet.

[90] Kim M.Y., Jung M., Noh Y., Shin S., Hong C.H., Lee S., Jung Y.S. (2020). Impact of Statin Use on Dementia Incidence in Elderly Men and Women with Ischemic Heart Disease. Biomedicines.

[91] Golomb B.A., Dimsdale J.E., Koslik H.J., Evans M.A., Lu X., Rossi S., Mills P.J., White H.L., Criqui M.H. (2015). Statin Effects on Aggression: Results from the UCSD Statin Study, a Randomized Control Trial. PLoS ONE.

[92] Schooling C.M., Au Yeung S.L., Freeman G., Cowling B.J. (2013). The Effect of Statins on Testosterone in Men and Women, a Systematic Review and Meta-Analysis of Randomized Controlled Trials. BMC Med..

[93] Bagchi S., Genardi S., Wang C.-R. (2018). Linking CD1-Restricted T Cells with Autoimmunity and Dyslipidemia: Lipid Levels Matter. Front. Immunol..

[94] Andersen C.J. (2018). Impact of Dietary Cholesterol on the Pathophysiology of Infectious and Autoimmune Disease. Nutrients.

[95] Ito A., Hong C., Oka K., Salazar J.V., Diehl C., Witztum J.L., Diaz M., Castrillo A., Bensinger S.J., Chan L. (2016). Cholesterol Accumulation in CD11c+ Immune Cells Is a Causal and Targetable Factor in Autoimmune Disease. Immunity.

[96] Artola R.T., Mihos C.G., Santana O. (2016). Effects of Statin Therapy in Patients with Systemic Lupus Erythematosus. South. Med. J..

[97] Li G., Zhao J., Li B., Zhang X., Ma J., Ma X., Liu J. (2018). The Anti-Inflammatory Effects of Statins on Patients with Rheumatoid Arthritis: A Systemic Review and Meta-Analysis of 15 Randomized Controlled Trials. Autoimmun. Rev..

[98] Budoff M. (2016). Triglycerides and Triglyceride-Rich Lipoproteins in the Causal Pathway of Cardiovascular Disease. Am. J. Cardiol..

[99] Group A.S. (2010). Effects of Combination Lipid Therapy in Type 2 Diabetes Mellitus. N. Engl. J. Med..

[100] D’Emden M.C., Jenkins A.J., Li L., Zannino D., Mann K.P., Best J.D., Stuckey B.G.A., Park K., Saltevo J., Keech A.C. (2014). Favourable Effects of Fenofibrate on Lipids and Cardiovascular Disease in Women with Type 2 Diabetes: Results from the Fenofibrate Intervention and Event Lowering in Diabetes (FIELD) Study. Diabetologia.

[101] Heron M. (2019). Deaths: Leading Causes for 2017. National Vital Statistics Reports.

[102] Huebschmann A.G., Huxley R.R., Kohrt W.M., Zeitler P., Regensteiner J.G., Reusch J.E.B. (2019). Sex Differences in the Burden of Type 2 Diabetes and Cardiovascular Risk across the Life Course. Diabetologia.

[103] Mauvais-Jarvis F. (2018). Gender Differences in Glucose Homeostasis and Diabetes. Physiol. Behav..

[104] Kautzky-Willer A., Harreiter J., Pacini G. (2016). Sex and Gender Differences in Risk, Pathophysiology and Complications of Type 2 Diabetes Mellitus. Endocr. Rev..

[105] Kautzky-Willer A., Harreiter J. (2017). Sex and Gender Differences in Therapy of Type 2 Diabetes. Diabetes Res. Clin. Pract..

[106] Franconi F., Campesi I. (2014). Sex and Gender Influences on Pharmacological Response: An Overview. Expert Rev. Clin. Pharmacol..

[107] Dennis J.M., Henley W.E., Weedon M.N., Lonergan M., Rodgers L.R., Jones A.G., Hamilton W.T., Sattar N., Janmohamed S., Holman R.R. (2018). Sex and BMI Alter the Benefits and Risks of Sulfonylureas and Thiazolidinediones in Type 2 Diabetes: A Framework for Evaluating Stratification Using Routine Clinical and Individual Trial Data. Diabetes Care.

[108] Ilias I., Rizzo M., Zabuliene L. (2022). Metformin: Sex/Gender Differences in Its Uses and Effects—Narrative Review. Medicina.

[109] De Vries S.T., Denig P., Ekhart C., Mol P.G.M., van Puijenbroek E.P. (2020). Sex Differences in Adverse Drug Reactions of Metformin: A Longitudinal Survey Study. Drug Saf..

[110] Li J., Li J., Shan Z., Yang W., Liu J., Tian H., Zhou Z., Ji Q., Weng J., Jia W. (2021). Gender-Differential Effects on Blood Glucose Levels between Acarbose and Metformin in Chinese Patients with Newly Diagnosed Type 2 Diabetes: A Sub-Analysis of the March Trial. Endocr. J..

[111] Wang Y., Xiao J., Zhao Y., Du S., Du J. (2020). Effect of Metformin on the Mortality of Colorectal Cancer Patients with T2DM: Meta-Analysis of Sex Differences. Int. J. Colorectal Dis..

[112] Raparelli V., Elharram M., Moura C.S., Abrahamowicz M., Bernatsky S., Behlouli H., Pilote L. (2020). Sex Differences in Cardiovascular Effectiveness of Newer Glucose-Lowering Drugs Added to Metformin in Type 2 Diabetes Mellitus. J. Am. Heart Assoc..

[113] Wang S.H., Chen W.J., Hsu L.Y., Chien K.L., Wu C.S. (2018). Use of Spontaneous Reporting Systems to Detect Host-Medication Interactions: Sex Differences in Oral Anti-Diabetic Drug-Associated Myocardial Infarction. J. Am. Heart Assoc..

[114] Kosi-Trebotic L., Thomas A., Harreiter J., Chmelik M., Trattnig S., Kautzky-Willer A. (2017). Gliptin Therapy Reduces Hepatic and Myocardial Fat in Type 2 Diabetic Patients. Eur. J. Clin. Investig..

[115] Zhao X., Wang M., Wen Z., Lu Z., Cui L., Fu C., Xue H., Liu Y., Zhang Y. (2021). GLP-1 Receptor Agonists: Beyond Their Pancreatic Effects. Front. Endocrinol..

[116] Rentzeperi E., Pegiou S., Koufakis T., Grammatiki M., Kotsa K. (2022). Sex Differences in Response to Treatment with Glucagon-like Peptide 1 Receptor Agonists: Opportunities for a Tailored Approach to Diabetes and Obesity Care. J. Pers. Med..

[117] Yang R.-Z., Lee M.-J., Hu H., Pray J., Wu H.-B., Hansen B.C., Shuldiner A.R., Fried S.K., McLenithan J.C., Gong D.-W. (2006). Identification of Omentin as a Novel Depot-Specific Adipokine in Human Adipose Tissue: Possible Role in Modulating Insulin Action. Am. J. Physiol. Metab..

[118] De Souza Batista C.M., Yang R.-Z., Lee M.-J., Glynn N.M., Yu D.-Z., Pray J., Ndubuizu K., Patil S., Schwartz A., Kligman M. (2007). Omentin Plasma Levels and Gene Expression Are Decreased in Obesity. Diabetes.

[119] Pan H.-Y., Guo L., Li Q. (2010). Changes of Serum Omentin-1 Levels in Normal Subjects and in Patients with Impaired Glucose Regulation and with Newly Diagnosed and Untreated Type 2 Diabetes. Diabetes Res. Clin. Pract..

[120] Tan B.K., Adya R., Farhatullah S., Lewandowski K.C., O’Hare P., Lehnert H., Randeva H.S. (2008). Omentin-1, a Novel Adipokine, Is Decreased in Overweight Insulin-Resistant Women with Polycystic Ovary Syndrome: Ex Vivo and In Vivo Regulation of Omentin-1 by Insulin and Glucose. Diabetes.

[121] Seufert J. (2004). Leptin Effects on Pancreatic β-Cell Gene Expression and Function. Diabetes.

[122] Esteghamati A., Noshad S., Rabizadeh S., Ghavami M., Zandieh A., Nakhjavani M. (2013). Comparative Effects of Metformin and Pioglitazone on Omentin and Leptin Concentrations in Patients with Newly Diagnosed Diabetes: A Randomized Clinical Trial. Regul. Pept..

